# Flavonoids from *Gynostemma pentaphyllum* Exhibit Differential Induction of Cell Cycle Arrest in H460 and A549 Cancer Cells

**DOI:** 10.3390/molecules191117663

**Published:** 2014-10-31

**Authors:** Ko-Chung Tsui, Tzu-Hsuan Chiang, Jinn-Shyan Wang, Li-Ju Lin, Wei-Chih Chao, Bing-Huei Chen, Jyh-Feng Lu

**Affiliations:** 1 School of Medicine, Fu Jen Catholic University, New Taipei City 24205, Taiwan; E-Mails: tsuiko@hotmail.com (K.-C.T.); fjmedwang@gmail.com (J.-S.W.); a9042020@gmail.com (L.-J.L.); tootol@hotmail.com (W.-C.C.); 2 Department of Internal Medicine, Cathay General Hospital, Taipei 10630, Taiwan; 3 Graduate Institute of Medicine, Fu Jen Catholic University, New Taipei City 24205, Taiwan; E-Mail: flights_2@hotmail.com; 4 Department of Food Science, Fu Jen Catholic University, New Taipei City 24205, Taiwan; E-Mail: 002622@mail.fju.edu.tw

**Keywords:** *Gynostemma pentaphyllum*, flavonoid, lung cancer cell, apoptosis

## Abstract

Flavonoids, containing mainly kaempferol rhamnohexoside derivatives, were extracted from *Gynostemma pentaphyllum* (*G. pentaphyllum*) and their potential growth inhibition effects against H460 non-small cell lung cancer cells was explored and compared to that on A549 cells. The extracted flavonoids were found to exhibit antiproliferation effects against H460 cells (IC_50_ = 50.2 μg/mL), although the IC_50_ of H460 is 2.5-fold that of A549 cells (IC_50_ = 19.8 μg/mL). Further investigation revealed that H460 cells are more susceptible to kaempferol than A549, whereas A549 cell growth is better inhibited by kaempferol rhamnohexoside derivatives as compared with H460. In addition, flavonoids from *G. pentaphyllum* induced cell cycle arrest at both S and G2/M phases with concurrent modulated expression of the cellular proteins cyclin A, B, p53 and p21 in A549 cells, but not H460. On the contrary, apoptosis and concomitant alteration in balance of BCL-2 and BAX expression as well as activation of caspase-3 were equally affected between both cells by flavonoid treatment. These observations strongly suggest the growth inhibition discrepancy between H460 and A549 following flavonoid treatment can be attributed to the lack of cell cycle arrest in H460 cells and the differences between H460 and A549 cells may serve as contrasting models for further mechanistic investigations.

## 1. Introduction

Numerous natural compounds have been extensively investigated for their potential in cancer therapy and prevention over the decades. Based on a recent review of natural products as sources of new drugs from 1981 to 2012, a total of 44 (~45%) out of 99 new anti-cancer drugs are either natural products or their derivatives [1]. Paclitaxel (Taxol^®^), for example, is a naturally occurring diterpenoid isolated from the bark of the Pacific yew tree, *Taxus brevifolia*. It is demonstrated to be an inhibitor of cell division by interfering normal microtubule breakdown during mitosis and is currently used as anti-mitotic chemotherapy for lung, breast, ovarian, head and neck cancer, as well as advanced forms of Kaposi’s sarcoma [2].

*Gynostemma pentaphyllum* (*G. pentaphyllum*), also known as “Jiaogulan”, is commonly referred to the poor’s ginseng and is widely used as a folk medicine and herbal tea in Asian countries, including Taiwan, China, Japan, and Korea. Several vital biological activities including anti-cancer [3,4,5,6,7,8,9] anti-inflammation [10,11,12], anti-oxidation [13,14], anti-hyperglycemic [15,16,17] and anti-atherosclerosis effects [15,18,19,20] have been associated with intake of *G. pentaphyllum*. The major bioactive components of *G. pentaphyllum* in order of abundance include saponins, flavonoids, chlorophylls, and carotenoids. Among them, saponins and flavonoids are believed to be responsible for the beneficial properties of *G. pentaphyllum*. More than 165 dammarane triterpene saponins, also known as gypenosides, have been identified from *G. pentaphyllum* and four (gypenoside III, IV, VIII and XII) of them are identical to and are more abundant in amounts than those found in *Panax ginseng* (ginsenoside Rb_1_, Rb_3_, Rd and F_2_, respectively) [21]. Flavonoids, also referred to as vitamin P, are the most common polyphenolic plant compounds. They are derivatives with a basic structure of two benzene rings and a pyrene ring and can be divided into five subgroups—flavonols, flavones, flavonones, flavan-3-ols and anthocyanidins [22]. Flavonoids extracted from *G. pentaphyllum* mainly consist of kaempferol and quercetin derivatives.

The antiproliferation effect of saponins and flavonoids from *G. pentaphyllum* has been reported for a variety of cancer cell lines, including hepatoma [4,6,23], colorectal [24,25], prostate [8], leukemia [26], glioma [3], oral [7,27], tongue [28], esophageal [24] and lung cancer [9,29]. However, the majority of reports mainly focus on the effect of the saponins rather than the flavonoids from *G. pentaphyllum*. We previously reported that both saponins and flavonoids extracted from *G. pentaphyllum* were equally effective in suppressing the growth of prostate cancer PC-3 cells, with IC_50_ values of 39.3 and 33.3 μg/mL, respectively, by inducing cell cycle arrest at both S and G2/M phases as well as apoptosis [8]. Although the growth inhibition effect of saponins from * G. pentaphyllum* on lung cancer A549 cells via induction of both G0/G1 arrest and apoptosis has been observed and reported [29], the effects of flavonoids from *G. pentaphyllum* on the growth of A549 and other lung cancer cells have never been examined. In addition, flavonoids from different sources have been reported to exhibit antiproliferation effects against lung cancer cell lines. For example, flavonoids isolated from Korean *Citrus aurantium* L. were reported to suppress the growth of A549 cells through G2/M arrest and the induction of apoptosis [30]. The herbal flavonoid quercetin was also shown to inhibit the growth of H460 cells by inducing apoptosis via the NF-κB pathway [31].

Based on the GLOBOCAN 2008 report from the International Agency for Research on Cancer, lung cancer is the most common cause of cancer-related deaths in men and women worldwide. The main types of lung cancer include small-cell lung carcinoma (SCLC; 15%), and non-small-cell lung carcinoma (NSCLC; 85%) which is further subdivided into adenocarcinoma, squamous, and large cell carcinoma [32]. Although limited options of targeted therapy for lung cancer have been developed and available for clinical use, including the tyrosine kinase inhibitors gefitinib (Iressa^®^), erlotinib (Tarceva^®^), and crizotinib (Xalkori^®^), the need for controlling disease progression among certain patients with few side effects warrants the further search for molecules with defined intervening targets and therapeutic benefits in cancer treatment. To explore the potential use of flavonoids from *G. pentaphyllum* as a therapeutic adjuvant for lung cancer, two NSCLC cell lines, H460 and A549, were utilized as cell models to evaluate the growth inhibition efficacy and to elucidate the molecular mechanism(s) involved in the antiproliferation effects of flavonoids on these cells.

## 2. Results and Discussion

### 2.1. HPLC-MS Analysis of Flavonoids from G. pentaphyllum

*G. pentaphyllum* has been investigated previously as a potential source of natural compounds with antiproliferation effects on cancer cells [4,7,13] or for alleviating cardiovascular disease [19,20] and diabetes [16]. We have previously identified and reported a total of 17 saponins and nine flavonoids extracted from *G. pentaphyllum* using Cosmosil 75C_18_ open column chromatography followed by HPLC-MS analysis [8]. The major components of flavonoids from *G. pentaphyllum* and their relative abundance are summarized in Table 1. The top four most abundant components, including kaempferol rhamnohexoside (64.2%), quercetin rhamnohexoside (20.4%), rutin (6.6%) and quercetin-di-(rhamnohexoside) (6.1%) comprised more than 97% of the extracted flavonoids. 

**Table 1:** Major components and relative contents (%) of flavonoids from *G. pentaphyllum* [8].

Fraction	Components	Content Percentage (%) [8]
Flavonoids		
	Kaempferol rhamnohexoside	64.2%
	Quercetin rhamnohexoside	20.4%
	Rutin	6.6%
	Quercetin-di-(rhamnohexoside)	6.1%
	Kaempferol-3-O-rutinoside	1.7%

### 2.2. Antiproliferation Effects of Flavonoids on H460 and A549

**Figure 1:**
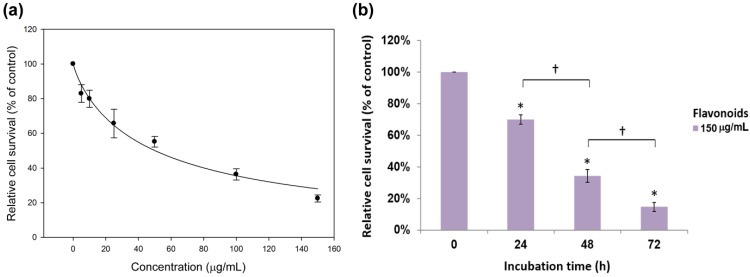
(**a**) Inhibition effect of flavonoids from *G. pentaphyllum* on H460 cell growth as determined by MTT assay. Data are shown as averages with indicated standard deviations (*n* = 3). The estimated IC_50_ of the flavonoid fraction is 50.2 μg/mL; (**b**) Inhibition effect of 150 μg/mL flavonoid fractionon H460 cells as affected by incubation time. Data are shown as averages with indicated standard deviations (*n* = 4). Data with asterisks (*) and dagger (†) indicate statistic significant (*p* < 0.05) as compared with control and between two groups, respectively.

Based on a large survey from 2006 to 2010, lung cancer is the leading cause of cancer-related death and the third most common cancer in the United State [32]. More than 85% of lung cancer cases are non-small cell carcinoma, among which adenocarcinoma is the most common subtype, accounting for more than 40% of all lung cancers. We have previously reported that both saponins and flavonoids extracted from *G. pentaphyllum* exhibited antiproliferation effects against the human prostate cancer cell line PC-3 via induction of cell cycle arrest and apoptosis [8]. In the present study, we utilized a non-small cell lung cancer (NSCLC) cell line H460, derived from large cell carcinoma of lung cancers, as a study model to explore the potential use of flavonoids from *G. pentaphyllum* as therapeutic adjuvant for lung cancer and to study their molecular mechanism of action. Figure 1a shows the inhibition effect of flavonoids from *G. pentaphyllum* on H460 cell growth as determined by the MTT assay. An apparent dose-dependent growth inhibition on H460 cells was observed over the doses up to 150 μg/mL for flavonoid. With flavonoid dose at 50 μg/mL, the H460 cell survival rate was 55%, followed by a dose-dependent decline and a 78% inhibition was attained at 150 μg/mL. The estimated half maximal inhibitory concentration (IC_50_) of flavonoid fraction was determined to be 50.2 μg/mL.

Since the flavonoid fraction from *G. pentaphyllum* exhibited inhibitory effects against H460 cells, the effect of the flavonoid fraction on inhibition of H460 cells as affected by incubation time was investigated and is shown in Figure 1b. At a dose of 150 μg/mL, a time-dependent inhibition on H460 cells was observed over an incubation period of 24–72 h. Specifically, 30%, 66%, and 85% of H460 cells were growth inhibited by incubation with the flavonoid fraction from *G. pentaphyllum* for 24, 48 and 72 h, respectively.

**Figure 2:**
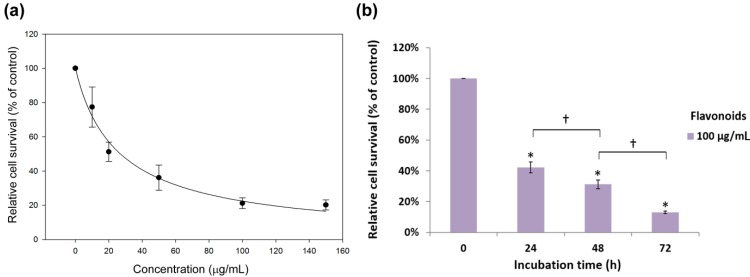
(**a**) Inhibition effect of the flavonoid fraction from *G. pentaphyllum* on A549 cell growth as determined by MTT assay. The estimated half maximal inhibitory concentration (IC_50_) is 19.8 μg/mL; (**b**) Inhibition effect of 100 μg/mL flavonoid fraction on A549 cells as affected by incubation time. Data are shown as averages with indicated standard deviations (*n* = 3). Data with asterisks (*) and daggers (†) indicate statistic significant (*p* < 0.05) as compared with control and between two groups, respectively.

To examine whether flavonoids are effective against other non-small cell lung cancers, A549, another commonly used NSCLC cell line derived from adenocarcinoma lung cancer, was utilized for comparison. As shown in Figure 2a, the flavonoid fraction from *G. pentaphyllum* exhibited both dose- and time-dependent inhibition on the growth of A549 cells. At a concentration of 20 μg/mL, the average survival rate of A549 cells was 51%. When the concentration was increased to 100 μg/mL, the average survival rate of A549 was reduced to 21% and remained at 20% for 150 μg/mL. The IC_50_ was 19.8 μg/mL. As shown in Figure 2b, when the incubation time of 100 μg/mL flavonoids increased from 24 to 48 and 72 h, the cell survival rate of A549 declined from 42% to 31% and 13%, respectively. As a result, flavonoids from *G. pentaphyllum* are more effective against the growth of A549 than H460 as indicated by the IC_50_ for H460 (50.2 μg/mL) is more than 2.5-fold of that for A549.

**Figure 3:**
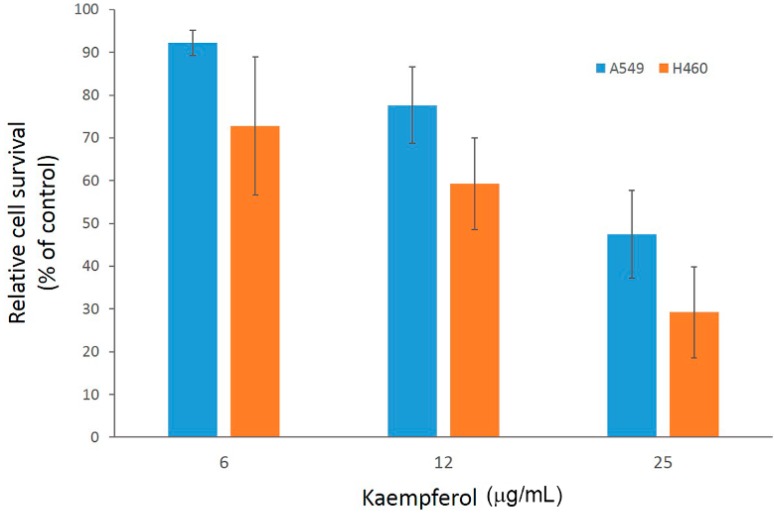
Inhibition effect of kaempferol on A549 and H460 cell growth as determined by an MTT assay. Data are shown as averages with indicated standard deviations of at least triplicate experiments.

Since flavonoids from *G. pentaphyllum* contains mainly kaempferol rhamnohexoside derivatives (64.2%; Table 1), the growth inhibition effect of kaempferol on A549 and H460 were also investigated for comparison. As shown in Figure 3, kaempferol does exhibit dose-dependent growth inhibition on both A549 and H460 cells. The relative cell survival in the presence of 6, 12 and 25 μg/mL kaempferol (equivalent to 21.8, 43.7 and 87.3 μM, respectively) after 72 h was 92%, 78% and 47% for A549 and 73%, 59% and 29% for H460 cells, respectively. Although a similar trend of inhibition was observed for both cells, unlike the case of flavonoids from *G. pentaphyllum*, kaempferol exerted slightly better inhibition on H460 than on A549 cells. These observations implied that growth inhibition efficacy of flavonoids varies among NSCLC cell types, among which H460 cells are more susceptible to kaempferol than A549, whereas A549 cell growth is better inhibited by kaempferol rhamnohexoside derivatives and/or other components in the fraction as compared with H460. This apparent discrepancy in antiproliferation effects also suggests possible divergence of cellular responses following flavonoid treatment in A549 *versus* H460 cells.

### 2.3. Cell Cycle Analysis

**Figure 4:**
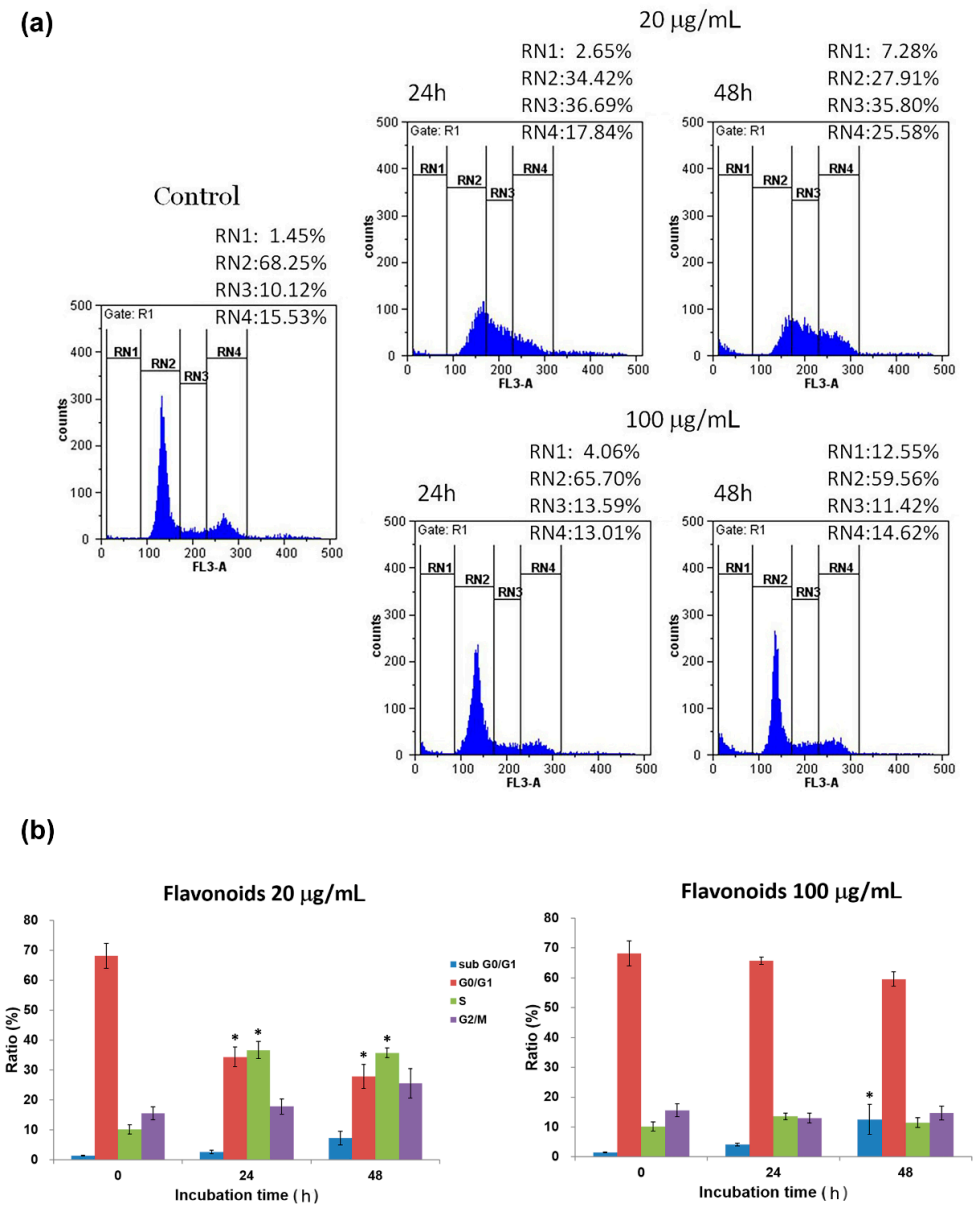
(**a**) Effects of 20 and 100 μg/mL flavonoidtreatment at indicated time intervalson cell cycle distribution of A549 cells. Cell population percentages of sub-G0/G1, G0/G1, S and G2/Mphases are indicated as RN1, RN2, RN3 and RN4%, respectively; (**b**) Statistical analyses are shown as averages with indicated standard errors (*n* = 3). Data with asterisks (*) indicate statistic significant (*p* < 0.05) as compared with control.

To investigate the molecular mechanism(s) involved in the observed growth inhibition, the cell cycles of A549 and H460 cells exposed to the flavonoids were examined. As shown in Figure 4, the pattern of distribution at different phases for A549 was significantly altered after incubation with 20 μg/mL of flavonoids for 24 and 48 h. An apparent reduction of A549 cells at G0/G1 was found with concomitant increases in both S and G2/M phases following flavonoid treatment. As summarized in Figure 4b, the G0/G1 cells decreased from 68.3% in control to 34.4% and 27.9% after 24 and 48 h of 20 μg/mL flavonoid treatment, whereas cells at S and G2/M phases increased from 10.1% and 15.5% in control, to 36.7% and 17.8% after 24 h and, to 35.8% and 25.6% after 48 h of flavonoid treatment, respectively. Similarly, a slight increase of sub G0/G1 cells was also observed from 1.5% in control to 2.7% and 7.3% after incubation for 24 and 48 h, respectively. At a dose of 100 μg/mL, a more significant increase in sub G0/G1 cells was found from 1.5% in control to 4.1 after 24 h and to 12.6% after 48 h of flavonoid treatment. However, no apparent signs of cell cycle arrest and only small but statistically insignificant variation were observed in all other phases of the cell cycles, suggesting both cell cycle arrest and apoptosis indeed occurred among A549 cells treated with flavonoids, especially around the dose of IC_50_ (about 20 μg/mL).

**Figure 5:**
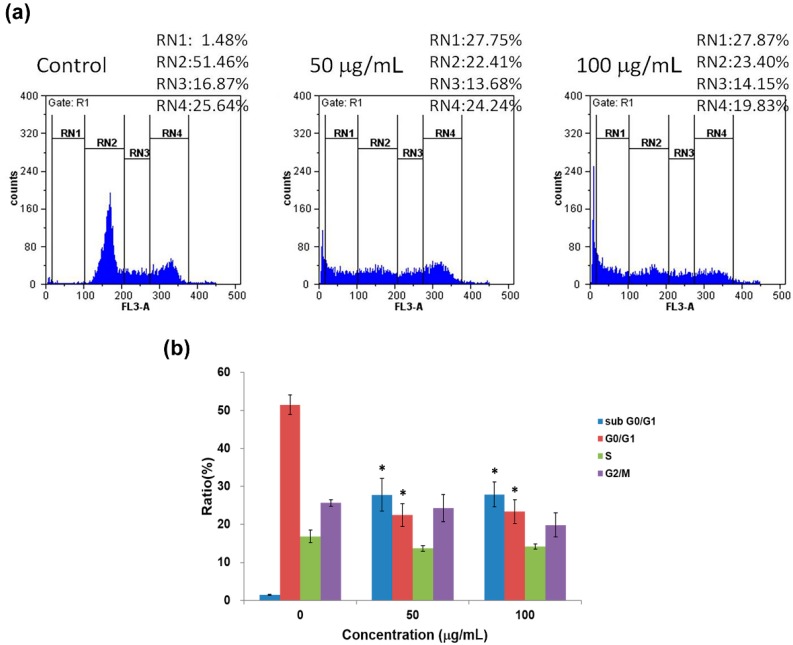
(**a**) Effects of 50 and 100 μg/mL flavonoid treatment for 48 hon cell cycle distribution of H460 cells. Cell population percentages of sub-G0/G1, G0/G1, S and G2/Mphases are indicated as RN1, RN2, RN3 and RN4%, respectively; (**b**) Statistical analyses are shown as averages with indicated standard errors (*n* = 3). Data with asterisks (*) indicate statistic significant (*p* < 0.05) as compared with control.

As seen in Figure 5, H460 cells exhibited even greater increase in sub G0/G1 ratio, from 1.5% in control to 27.8% and 27.9% following both 50 and 100 μg/mL of flavonoid treatment for 48 h, respectively, which were accompanied by parallel reduction of G0/G1 ratio from 52.5% in control to 22.4 and 23.4%. No obvious signs of arrest in both S and G2/M phases were found after treating H460 with either dose of flavonoids, in which S and G2/M ratios were 16.9% and 25.6% in control and remained at 13.7% and 14.2% as well as 24.2% and 20.0% after flavonoid treatment at 50 and 100 μg/mL, respectively. As a result, although the cell cycle of H460 was clearly disturbed, as more than half of the G0/G1 population shifted to the sub-G0/G1 phase, no apparent arrest at S or G2/M phase was detected, implying that apoptosis is possibly the predominating event in H460 cells following flavonoid treatment.

### 2.4. Apoptosis Analysis by Annexin-V/PI Staining Assay

**Figure 6:**
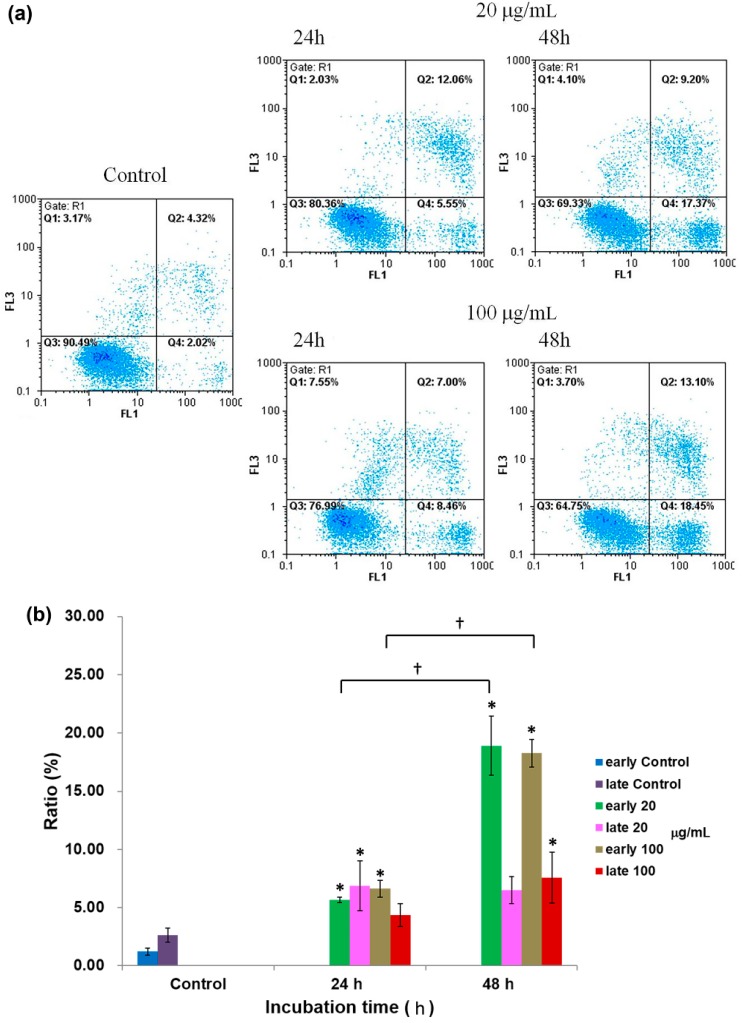
(**a**) Annexin-V/PI analysis by flow cytometry of A549 cells treated with 20 and 100 μg/mLflavonoidsfrom *G. pentaphyllum*. Q3, normal; Q4, annexin V positive (*i.e.*, early apoptotic population); Q1, PI positive; Q2, Annexin/PI positive (*i.e.*, late apoptotic population); (**b**) Statistical analyses are shown as averages with indicated standard errors (*n* = 4). Data with asterisks (*) and daggers (†) indicate statistic significant (*p* < 0.05) as compared with control and between two groups, respectively.

**Figure 7:**
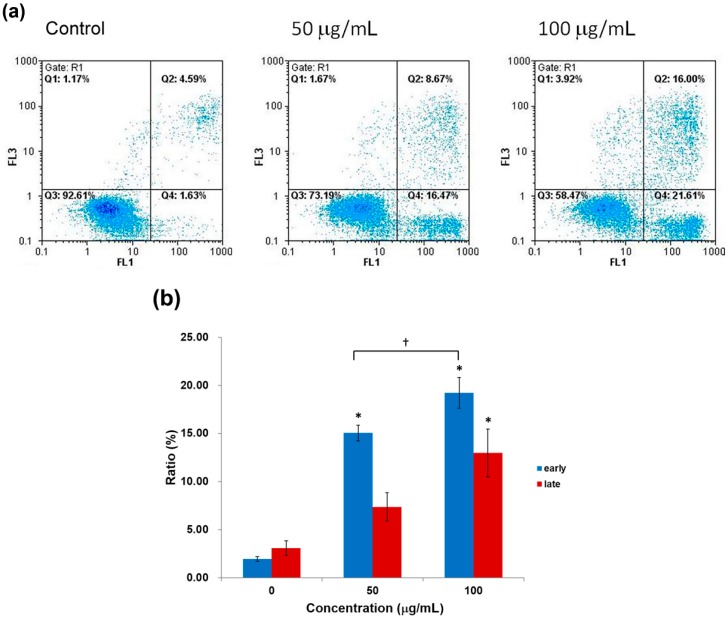
(**a**) Annexin-V/PI analysis by flow cytometry of H460 cells treated with 50 and 100 μg/mLflavonoids from *G. pentaphyllum*. Q3, normal; Q4, annexin V positive (*i.e.*, early apoptotic population); Q1, PI positive; Q2, Annexin/PI positive (*i.e.*, late apoptotic population); (**b**) Statistical analyses are shown as averages with indicated standard errors (*n* = 4). Data with asterisks (*) and daggers (†) indicate statistic significant (*p* < 0.05) as compared with control and between two groups, respectively.

The annexin-V/PI double staining assay further confirmed that apoptosis events indeed occurred in both A549 and H460 cells following flavonoid treatment. This double staining assay detects the presence of phosphatidylserine translocation to the outer leaflet of the plasma membrane in apoptotic cells (Figure 6 and Figure 7). During apoptosis, translocated phosphatidylserine conjugates with FTIC-labeled annexin-V to emit fluorescence that can be detected by FL1 channel. PI is a vital fluorescent dye, as measured by FL3 channel, and is used for the evaluation of cellular viability. Representative assay results for A549 cells are shown in Figure 6a, and statistical analyses are summarized in Figure 6b. Both early (Q4 quadrant) and late (Q2 quadrant) stages of apoptotic A549 cells increased in a time-dependent manner. At a dose of 20 μg/mL, the early and late apoptotic cells rose from 1.2% and 2.6% in control to 5.7% and 6.9% after 24 h and to 18.9% and 6.5% after 48 h incubation with flavonoids, respectively. A plateau of late apoptotic cell population was observed between 24 to 48 h after 20 μg/mL of flavonoid treatment. When flavonoids increased to 100 μg/mL, both early and late apoptotic cells increased to 6.6% and 4.3% after 24 h and, to 18.3% and 7.6% after 48 h treatment, respectively. In addition, both early and late apoptotic cell populations demonstrated a trend of dose-dependent increase by flavonoid treatment, with the exception of slight decrease in late apoptotic cell population treated with 20 (6.9%) *vs.* 100 (4.3%) μg/mL flavonois.

An even more significant apoptotic phenomenon was observed in H460 cells following flavonoid treatment at doses of 50 and 100 μg/mL for 48 h (Figure 7). As summarized in Figure 7b, early apoptotic H460 cells increased from 1.9% in control, to 15.0% and 19.2% after 50 and 100 μg/mL of flavonoid treatment, respectively. Similarly, late apoptotic cells also increased from 3.1% in control to 7.4% and 13.0% after treatment. As a result, both early and late apoptotic populations were significantly increased in both time- and concentration-dependent manners following flavonoid treatment.

### 2.5. Expression of Proteins Associated with Cell Cycle Control and Apoptosis

**Figure 8:**
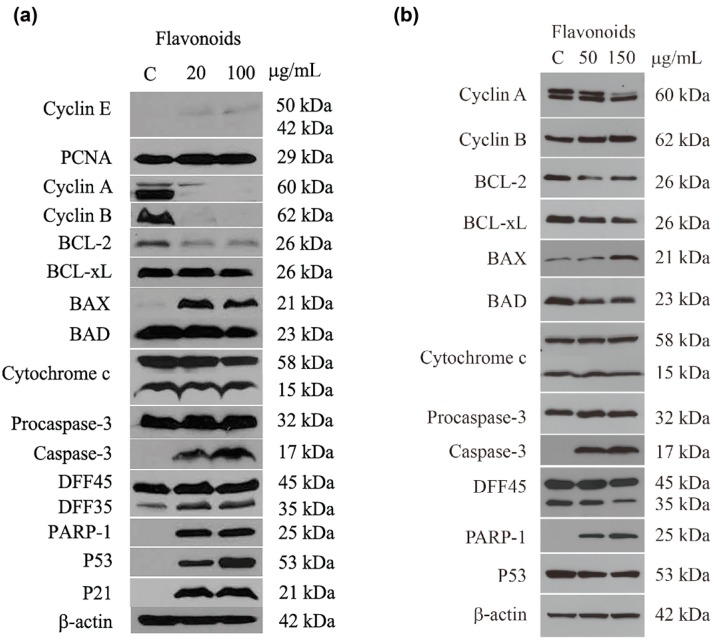
Expression of selected cellular proteins in A549 (**a**) and H460 (**b**) cells treated with flavonoids from *G. pentaphyllum*. β-actin was used as a protein loading control.

As described earlier, flavonoids induced arrest at S and G2/M phases in A549, as well as apoptosis in both A549 and H460 cells. To determine whether expression of relevant regulatory proteins was modulated, semi-quantitative western blotting was performed. The expression of proteins associated with cell cycle control and apoptosis in A549 and H460 cells treated with flavonoid fraction from *G. pentaphyllum* is shown in Figure 8. Similar intensity of β-actin signals in all lanes indicated equal amounts of proteins in control and flavonoid treated samples were loaded for analyses. Cyclin E regulates transition of G1 to S phase and PCNA is a marker for DNA replication in S phase. Both proteins exhibited minimal changes in amount in A549 cells with or without 20 and 100 μg/mL of flavonoid treatment for 36 h, even though the basal expression level of cyclin E was very low compared with that of PCNA in control. On the contrary, expressions of both cyclin A and B were high in control and diminished quickly following flavonoid treatment at both doses. The expressions of p53, a tumor suppressor and an initiator for both cell cycle arrest and apoptosis [33], and its downstream target p21 were also significantly induced after both 20 and 100 μg/mL of flavonoid treatment (Figure 8a). These results are in agreement with our findings described above that a large portion of A549 cells arrested at S and G2/M phases after being treated with 20 µg/mL of flavonoids containing mainly kaempferol rhamnohexoside. A similar phenomenon has been reported for other plant flavonoids as well. For example, isoliquiritigenin, a natural flavonoid isolated from licorice, was shown to induce an arrest of A549 at G2/M phase, with concomitant increase in the expression of p21 [34]. Flavonoids isolated from Korean *Citrus aurantium* L. induced cell cycle arrest at the G2/M checkpoint by controlling expression of cyclin B1, cdc2, cdc25c and p21 [30]. Consistently, kaempferol (50–100 μM) was previously reported to induce an arrest of hepatic cancer cell (SK-HEP-1) at G2/M phase via modulation of CDK1/cyclin B expression and the AMPK and AKT signaling pathways [23]. Similar outcomes were also shown for the breast cancer MDA-MB-453 cell line treated with 10 and 50 μM kaempferol for up to 24 h [35]. In addition, a similar but to a much lesser extent of cell cycle shifting phenomenon along with significant modulation of cell cycle regulatory proteins, including cyclin A and B, as well as p53 and p21, was observed in A549 cells treated with 100 μg/mL flavonoids, suggesting cell cycle arrest was indeed in effect among A549 cells treated with flavonoids from *G. pentaphyllum*. Although similar modulation of p53 and p21 expression leading to both G2/M arrest and apoptosis has also been reported for A549 cells treated with 20–40 μM quercetin [36], an increase expression of cyclin B1, rather than a decrease as shown in Figure 8a, was observed.

The BCL protein family, including the anti-apoptotic regulators BCL-2 and BCL-xL as well as pro-apoptotic regulators BAX and BAD, coordinates signals for cell survival or apoptosis. In A549 cells, clear reduction in BCL-2 and induction in BAX expression were detected after both 20 and 100 μg/mL of flavonoid treatment. However, flavonoids had a very limited effect on the expression levels of both BCL-xL and BAD as well as cytochrome c, a mitochondrial membrane protein that translocates to cytsol during apoptosis. Imbalanced expression of BCL-2 and BAX by flavonoids led to activation of caspase-3 by cleavage of procaspase-3 in A549 cells in a dose-dependent manner. This activation of apopotosis executioner caspase resulted in degradation of its downstream substrates, DFF45 (45 kDa) and PARP-1 (116 kDa), into smaller products of 35 and 25 kDa, respectively. Consistent with this result, Nguyen *et al.* reported that kaempferol induced apoptosis and similarly modulated expression of BCL-2 family proteins in A549 cells at concentrations of 35 to 70 μM [37]. Quercetin at concentrations of 14.5 to 58 μM was also reported by the same group to induce apoptosis and to modulate expression of apoptotic markers in A549 cells in a similar way, resulting in activation of caspase-3 and its downstream effector PARP-1 for apoptosis [38]. However, expressions of BCL-xL and BAD were significantly altered than those by flavonoid treatment, indicating cellular responses to flavonoids and aglycones of A549 cells were potential different.

For H460 cells, as shown in Figure 8b, the main protein expression altered by flavonoids was regulators involved in apoptosis rather than in cell cycle control. In addition to similar expression modulations observed in A549 cells, including reduction of BCL-2, induction of BAX, and activation of both caspase-3 and PARP-1, a slight decrease in the expression of both BCL-xL and BAD was also found in H460 following flavonoid treatment. On the contrary, the expressions of cyclin A and B as well as p53 were not affected in H460 cells as observed in A549 cells in the presence of flavonoids from *G. pentaphyllum*. The lack of modulation in the expression of examined cyclins may contribute, at least in part, to the absence of G2/M arrest in H460 cells. Furthermore, both A549 and H460 cells exhibited comparable porportions of both early and late apoptotic cells after 48 h of treatment (Figure 6 and Figure 7) along with increased BAX as well as decreased BCL-2 expressions (reduction of BCL-xL only in H460) in a dose-dependent manner after flavonoid treatment, suggesting apoptosis induction by flavonoids in both cells is likely to proceed through similar mechanisms. It is well known that both A549 and H460 cells harbor wild-type p53 allele. Unlike in A549, the expression of cellular p53 was constitutive and was not significantly altered in H460 following flavonoid treatment. The p53 protein has been shown to mediate both cell cycle arrest and apoptosis [33], thus implying the apoptosis induced by flavonoids in H460 cells might also proceed through a p53-independent pathway. Similar observation has been reported for herbal flavonoid quercetin, derivatives of which is second abundant in flavonoids extracted from *G. pentaphyllum*, to increase sub-G0/G1 and apoptotic cell populations regardless of p53 status [31]. Both quercetin and kaempferol have also been shown to induce apoptosis in A549 cells via MEK-ERK pathway [37,38]. In addition, the IC_50_ of growth inhibition by flavonoids from *G. pentaphyllum* is 2.5-fold for H460 than that for A549 cells. As only A549 showed cell cycle arrest at S and G2/M following flavonoid treatment, it is reasonable to conclude that the difference in growth inhibition efficacy associated with flavonoid treatment is most likely attributed to differential effect on the induction of cell cycle arrest among these cells.

## 3. Experimental Section

### 3.1. Materials

*G. pentaphyllum* was acquired from a local herbal dealer in Taipei, Taiwan. Identification of *Gynostemma pentaphyllum (Thunb.) Makino* was confirmed by Dr. Bing-Huei Chen [4]. A voucher specimen has not been deposited, however, the dry processed sample is available upon request. HPLC-grade solvents, including methanol, acetonitrile, and acetone, were procured from Lab-Scan (Gliwice, Poland). Analytical-grade solvents, including hexane and ethanol, were from Lab-Scan and Grand Chemical (Taipei, Taiwan). Formic acid was from Riedel-de Häen Co. (Seelze, Germany). Deionized water was obtained by a Milli-Q water purification system from Millipore Co. (Bedford, MA, USA). The adsorbent Cosmosil 75C_18_-OPN, was from Nacalai Co. (Kyoto, Japan). Human lung adenocarcinoma A549 and large cell carcinoma H460 cell lines were from the Bioresource Collection and Research Center, Taiwan Food Industry Development and Research Institute/National Research Institute of Health (Hsinchu, Taiwan). RPMI-1640 medium, fetal bovine serum (FBS), penicillin-streptomycin, Trypan blue stain 0.4% solution and 2.5% trypsin-EDTA were from Life Technologies (Carlsbad, CA, USA). Ham’s F-12 medium, anti-β-actin, kaempferol (3,5,7-trihydroxy-2-(4-hydroxyphenyl)-4H-1-benzopyran-4-one) and 3(4,5-dimethylthiazolyl)-2,5-diphenyl-tetrazolium bromide (MTT) reagent were from Sigma (St. Louis, MO, USA). RNase A was from Roche (Basel, Switzerland). Sodium dodecyl sulfate (SDS), dimethyl sulfoxide (DMSO), Tween 20, 40% Acrylamide/Bis and TEMED were from J. T. Baker (Phillipsburg, NJ, USA). Propium iodide (PI) and Annexin-V were from BD Biosciences Co. (San Diego, CA, USA). The primary antibodies including anti-cyclin B, anti-DNA degradation factor 45 kDa (DDF45), anti-p21 and anti-cytochrome c were from BD Biosciences Co.; anti-caspase-3, anti-BCL-2, anti-BCL-xL, anti-BAD, anti-BAX, anti-poly [ADP-ribose] polymerase 1 (PARP-1) and anti-p53 were from Epitomics Co. (Burlingame, CA, USA). Anti-cyclin A and anti-proliferating cell nuclear antigen (PCNA) were from GeneTex Inc. (Irvine, CA, USA). Anti-cyclin E was from Thermo Fisher Scientific (Waltham, MA, USA). The secondary antibodies such as goat anti-mouse IgG-HRP and goat anti-rabbit IgG-HRP were from Santa Cruz Biotechnology Inc. (Dallas, TX, USA).

### 3.2. Extraction and Preparation of Flavonoids from G. pentaphyllum

Flavonoids were extracted from *G. pentaphyllum* with ethanol and purified by a column chromatographic method using Cosmosil 75C_18_-OPN as adsorbent [8]. Ethanol-water (30:70, v/v) eluted flavonoids were evaporated to dryness under vacuum, then redissolved in ethanol, filtered through a 0.22-μm membrane filter, and subjected to both qualitative and quantitative HPLC-MS analysis using a Gemini C_18_ column (Phenomenex Co.; Torrance, CA, USA) and a gradient mobile phase of 0.1% formic acid and methanol. HPLC-MS system was composed of an Agilent 1100 Series HPLC system (Agilent Technologies; Santa Clara, CA, USA), a column temperature controller, an on-line degasser, a photodiode-array detector, and a quadrupole mass spectrometer with multi-mode ion source.

### 3.3. MTT Assay

Human lung adenocarcinoma A549 and large cell carcinoma H460 cell lines were cultured in Ham’s F-12 and RPMI-1640 media, respectively, containing 5% FBS and 100 U/mL of penicillin-streptomycin, and were incubated under 5% CO_2_ at 37 °C. Replacement with fresh media was performed every two days to maintain optimal cell growth. When the cell density reaching 90%–95% confluency, cells were washed with PBS twice and detached from culture plates by treatment of 0.25% trypsin-EDTA. Cells were collected by centrifugation and resuspended in fresh medium. For MTT assay, 4 × 10^4^ A549 or 2 × 10^4^ H460 cells were seeded to each well in a 24-well culture plate to obtain approximately 50% confluency prior to treatments. The medium was replaced with fresh medium or medium containing indicated doses of extracted flavonoid fraction from *G. pentaphyllum* or kaempferol after 24 h incubation. After up to 72 h incubation, 50 μL of MTT reagent (5 mg/mL) was added to each well and incubated at 37 °C for 40 min, followed by adding 200 μL of DMSO to dissolve reduced formazan dye for 15–30 min. The resulting DMSO solution (150 μL) was collected to a 96-well plate and measured for absorbance at 570 nm with a Mulitiskan ELISA reader from Thermo (Fremont, CA, USA). The well with fresh medium without flavonoid extraction from *G. pentaphyllum* was used as control treatment. The relative cell survival rate was calculated based on the ratio of absorbance of flavonoid treatment over absorbance of control treatment for indicated time intervals.

### 3.4. Cell Cycle Analysis

A549 (1 × 10^6^) or H460 (5 × 10^5^) cells were seeded to a 10-cm culture plate and incubated for 24 h, the medium was then removed and replaced with fresh medium or medium containing indicated doses (20, 50 or 100 μg/mL) of flavonoid fraction. After up to 48 h incubation, cells were washed with PBS and detached by treatment with 0.25% trypsin-EDTA, followed by flushing with PBS. Cells were collected by centrifugation at 1200 rpm for 5 min and fixed with ice-cold 70% ethanol for 2 h, followed by centrifuging again at 1200 rpm for 5 min to remove fixative. Fixed cells were then washed with PBS for 3 times, and 500 μL of PI buffer, containing 0.1% Triton X-100, 2 μg/mL RNase A, and 20 μg/mL PI, was added for staining at 37 °C for 30 min. The cell cycle distribution of PI-stained cells was analyzed by a flow cytometer from Partec (Münster, Germany).

### 3.5. Annexin V and PI Staining Assay

A549 (1 × 10^6^) or H460 (5 × 10^5^) cells were seeded to a 10-cm plate and cultured for 24 h, after which indicated doses (20, 50 or 100 μg/mL) of flavonoid fraction were added and incubated for up to 48 h. Cells were then washed with PBS and detached by trypsin followed by neutralization with culture medium. The detached cells were collected into a 15-mL centrifuged tube, washed with ice-cold PBS twice, and centrifuged at 1200 rpm for 5 min to remove supernatant. A volume of 0.1 mL binding buffer, containing 0.14 M NaCl, 2.5 mM CaCl_2_ and 0.01 M Hepes/NaOH pH 7.4, was added to resuspend cells, followed by adding 5 μL of annexin V-FITC and 5 μL of 50 μg/mL PI staining reagents. After mixing homogeneously and reacting at 25 °C for 15 min in the dark, the apoptotic cell population was analyzed by a flow cytometer.

### 3.6. Western Blotting

A549 (1 × 10^6^) or H460 (5 × 10^5^) cells were cultured in a 10-cm plate and incubated for 24 h, after which the medium was removed and replaced with fresh medium or medium containing indicated doses (20, 50 and 100 μg/mL) of flavonoid fraction and incubated for another 36 h. Then the medium was removed followed by washing with PBS three times before detaching cells with a cell scraper and collecting by centrifuging at 1200 rpm for 5 min. Lysis buffer (Cell Signaling Technology, Danvers, MA, USA) was added to cell pellet of each treatment, and cells were disrupted with a sonicator for protein release, followed by centrifuging at 14,000 rpm (4 °C) for 10 min. The protein containing supernatant was collected and stored at −20 °C before use. Protein samples were quantified using Bio-Rad protein assay dye reagent and by absorbance measurement at 562 nm according to manufacturer’s instruction. The quantitative protein standard curve was prepared from serial dilution of standard bovine serum albumin solution (1 mg/mL) from Sigma.

Protein samples were prepared and adjusted to equal concentration in 1**×** SDS gel-loading buffer containing 50 mM Tris-HCl (pH 6.8), 0.1% bromophenol blue, 2% SDS, 5% β-mercaptoethanol and 10% glycerol, and subjected to protein separation on 10%, 12% or 15% SDS-polyacrylamide gel (SDS-PAGE) under 60 V for 30 min followed by 120 V for appropriate period. The separated protein was then transferred onto a nitrocellulose membrane in transfer buffer containing 0.04% SDS, 20% methanol, 40 mM glycine, and 50 mM Tris base pH 8.3 at 4 °C for 1 h. Nitrocellulose membrane was stained with Ponceau S and washed with deionized water for confirmation of protein transfer. Next, the nitrocellulose membrane was immersed in 5% skim milk at 25 °C for 20 min to remove background noise, followed by washing with TBST containing 0.05% Tween 20, 150 mM NaCl and 10 mM Tris-HCl pH 8.0 and adding the primary antibody for conjugation with target protein at 25 °C for 1 h. After washing with TBST 5 times for 5 min each, the horseradish peroxidase (HRP)-conjugated secondary antibody was added and incubated at 25 °C for 1 h, followed by washing 5 times with TBST for 5 min each. The SuperSignal West Dura Luminol/Enhancer (Thermo Fisher Scientific) was then added to produce chemiluminescence which was detected using autoradiograph film (Midwest Scientific, Valley Park, MO, USA) for protein expression signal and intensity measurement.

### 3.7. Statistical Analysis

All analyses were conducted at least in triplicate and the data were analyzed based on ANOVA and Duncan’s multiple range test to compare means for significant difference (*p* < 0.05) by employing a SPSS software system (IBM, New York, NY, USA).

## 4. Conclusions

In conclusion, the flavonoid fraction, containing mainly kaempferol rhamnohexoside derivatives, prepared from *G. pentaphyllum* was efficient in suppressing the growth of H460 and A549 NSCLC cells, by inducing an arrest of cell cycle at both S and G2/M phases in the latter, as well as apoptosis in both cells. A dose-dependent decrease in expression of the anti-apoptotic protein BCL-2 was shown for both cells, whereas a reverse trend was found for the expression of pro-apoptotic protein BAX, as well as caspase-3 and its downstream substrate PARP-1. In addition, abolished cellular expression of cyclin A and B, as well as induction of p53 and p21 were also observed only in A549 cells, and not H460. All these findings suggest that the lack of cell cycle arrest in H460 cells following treatment of kaempferol based flavonoids from *G. pentaphyllum* may lead to reduced growth inhibition efficacy as compared with that of A549 cells. This discrepancy in flavonoid treatment response among A549 and H460 cells may serve as contrasting models for further mechanistic investigation.
